# Prevalence of Depression and Anxiety Symptoms Among Patients With Cancer in Najran, Saudi Arabia

**DOI:** 10.7759/cureus.54349

**Published:** 2024-02-17

**Authors:** Mohamed A Ghowinam, Ammar A Albokhari, Ahmed M Badheeb, Mohamed Lamlom, Mari Alwadai, Aseel Hamza, Ali Aladalah

**Affiliations:** 1 Psychiatry, Al-Azhar University, Cairo, EGY; 2 Psychiatry, Hera General Hospital, Makkah, SAU; 3 Oncology, King Khalid Hospital-Oncology Center, Najran, SAU; 4 Psychiatry, Eradah Complex and Mental Health Services, Najran, SAU; 5 Psychiatry, Eradah Complex and Mental Health Services, Jeddah, SAU

**Keywords:** najran, saudi arabia, cancer, anxiety, depression

## Abstract

Background: Depression and anxiety are associated with poor health consequences in patients with cancer, and these mental health issues may affect cancer treatment. They are frequently triggered by stress, and cancer is among the most stressful conditions experienced by a person. Depression and anxiety are related to several sociodemographic variables in patients with cancer. However, only a few studies have examined the prevalence of depression and anxiety symptoms in patients with cancer in Saudi Arabia.

Objectives: To detect the prevalence of depression and anxiety symptoms among patients with cancer at the Najran Oncology Center in Najran, Saudi Arabia, and determine the risk factors associated with these symptoms.

Methods: A cross-sectional study was conducted from April 1, 2023, to September 30, 2023, on a convenience sample of patients diagnosed with cancer who were receiving chemotherapy at Najran Oncology Center, King Khalid Hospital, Najran, Saudi Arabia, and who agreed to participate in the study. The Hospital Anxiety and Depression Scale was used. Data on the demographic characteristics of the patients were collected using a self-administered questionnaire. Moreover, medical data were collected from the medical records of the patients.

Results: In total, 92 patients with various cancer diagnoses were recruited in this study. Among them, 51 and 41 were women and men, respectively. Moreover, 81 were married and 11 were either single, widow, or divorced. The mean age of the participants was 51.24 ± 15.15 years. The prevalence rates of depression and anxiety were 42.4% and 23.9%, respectively. There were significant associations between depression and marital status in patients with cancer-associated pain and those with a current history of chemotherapy. Furthermore, the association between anxiety and cancer-associated pain was significant. However, marital status was not associated with anxiety. Age, sex, family history of mental disorders, cancer duration, current cancer stage, and surgical interventions were not associated with the prevalence of depression and anxiety.

Conclusions: Our findings underline the importance of identifying depression and anxiety in patients with cancer. Marital status, presence of pain, and current chemotherapy history were significantly associated with depression and pain with anxiety.

Recommendations: Further studies with a higher number of patients with cancer should be conducted in Saudi Arabia and other Arab countries. Screening for depression and anxiety symptoms should be a part of the comprehensive evaluation of patients with cancer. Appropriate treatment interventions must be provided to patients with cancer who present with mental disorders.

## Introduction

Cancer is a common cause of mortality worldwide and a major barrier to increasing the average lifespan of people. According to the 2019 World Health Organization report, cancer is the primary or secondary cause of mortality in 112 of 183 nations. Furthermore, it is the third or fourth cause of mortality in 23 countries. According to data from the International Agency for Research on Cancer, there were approximately 18 million new cancer cases and 9 million cancer-related mortalities in 2018 alone [1]. Numerous risk factors, such as tobacco and alcohol use, poor diet, inactivity, bacterial and viral infections, indoor smoking, ionizing radiation, and urban air pollution, are associated with cancer etiology [2].

Due to changes in the demography of the population over the last decades, the number of cancer-related deaths is expected to continuously increase, reaching 21.4 million globally by 2030 [3]. In Saudi Arabia, there were 24,485 new cancer cases and 10,518 cancer-related deaths in 2018 (total population = 33,554,333) [4]. According to a recent study, the most common types of cancers in Saudi Arabia are breast, colorectal, prostate, brain, lymphoma, renal, and thyroid cancers [5]. Other studies have reported that the incidence of colorectal cancer in Saudi Arabia is increasing [6,7]. In Saudi Arabia, cancers are commonly associated with ambient air pollution because of the country’s regular exposure to dust storms that carry particulate matter [8].

Mental disorders are common among patients with cancer and are associated with unfavorable health outcomes [9]. Psychological distress affects 30-50% of patients with cancer [10]. Patients with cancer frequently experience anxiety and depression. Anxiety and depression are commonly triggered by stress, and cancer is among the most stressful conditions experienced by a person. Cancer treatment can be affected by these mental health conditions [11]. The presence of anxiety and depression may be associated with less successful treatment outcomes, longer length of hospital stays, and functional issues [12]. During illness, depression or anxiety affects more than one in four patients with cancer and requires medical and psychological care [13]. Up to 30% of patients with cancer have clinically significant depression symptoms, thereby making it a prevalent disease symptom [14]. According to a previous study, 28% of patients with cancer had depression [15]. Another study found that 32.9% of hospitalized patients with cancer had depression [16]. A study using the Hospital Anxiety and Depression Scale (HADS) found that 29% of patients with cancer presented with depression. In the present study, no difference was observed in terms of age, sex, and educational level. Furthermore, there was no difference in the nature and severity of pain and the level of disability between patients with depression and those without depression [17].

Anxiety is also prevalent among patients with cancer, and these patients should be identified and treated initially by cancer healthcare professionals [18]. Anxiety is associated with several variables, including sociodemographic functional status and social interactions, in patients with cancer [12]. A positive association has been reported between anxiety and increased side effects during chemotherapy [19]. According to a previous study, 18.5% of hospitalized patients with cancer presented with anxiety. Women were more likely to experience anxiety than men [16]. A multicenter study found that the prevalence of anxiety was 13.8% and that patients with cancer had a 2.7-fold higher risk of anxiety than the general population [19].

Only a limited number of studies have focused on the prevalence of depression and anxiety symptoms among patients with cancer in Saudi Arabia. Therefore, the present study aimed to assess depression and anxiety among these patients. Furthermore, it assessed the quality of health services delivered to patients with cancer in Saudi Arabia. In particular, the prevalence of depression and anxiety symptoms among patients with cancer and their associated risk factors were examined.

## Materials and methods

A cross-sectional study was conducted for six months (from April 1, 2023 to September 30, 2023). This study was approved by the Scientific Research Ethics Committee of King Khalid Hospital, Najran, Saudi Arabia (IRB registration number with KACST, KSA: H-l1-N-081). This study was conducted on a convenience sample of patients with cancer who were receiving chemotherapy at Najran Oncology Center, King Khalid Hospital, Najran, Saudi Arabia, and who agreed to participate. Two questionnaires were used. First, a self-administered questionnaire was used to collect data on the demographic characteristics of the participants (age, sex, marital status, and family history of mental disorder). Second, an Arabic version of the HADS, which is a four-point 14-item scale [20], was used. It aims to provide clinicians with an acceptable, reliable, valid, and easy-to-use practical tool for identifying and quantifying depression and anxiety [11].

It has two subscales for anxiety (seven items) and depression (seven items). For each item, the participants were instructed to indicate which of the four options (rated from 3 to 0; score range: 0-42) were the closest to describing how they have been feeling in the last week. A score of 0-7 indicated the absence of clinical symptoms of anxiety or depression; 8-10, mild anxiety or depression; and 11-21, symptomatic anxiety or depression. HADS was developed for populations with medical illness and was validated for older individuals and those with chronic pain [21-23]. An Arabic version of HADS has been used since 1987. Moreover, its application was validated for hospitalized patients in primary-care settings in Saudi Arabia [24-26]. Other medical data were collected from the medical records of the patients (cancer duration [months], cancer-associated pain, current cancer stage, current treatment, and history of surgical intervention for cancer).

The inclusion criteria were as follows: Arabic-speaking patients with cancer at Najran Oncology Center, King Khalid Hospital, Najran, Saudi Arabia. The cohort included patients with various cancer stages, those with different cancer durations, and males and females without discrimination. The exclusion criteria were patients aged 18 years, those with a history of mental illness, those receiving psychotropic medications, those with a history of substance use, and those with mental retardation.

Data were transferred from an Excel spreadsheet to the Statistical Package for the Social Sciences software (version 25.0, IBM Corp., Armonk, NY). Then, they were processed, analyzed, and presented in tables and figures. For descriptive statistics, mean ± standard deviation and median (interquartile range [IQR]) were used for quantitative variables, and frequency and percentage for qualitative variables. The numerical data were examined for normality using the Shapiro-Wilk test. The Fisher’s exact test or chi-square test was used to assess differences in the frequencies of qualitative variables. The specific association between depression and anxiety scores and different study variables was further explored. Both crude and adjusted odds ratios (ORs) with 95% confidence intervals (95% CIs) were considered using three models: model I (referred to as unadjusted) including the total sample, model II (adjusted for sociodemographic characteristics and family history of mental disorders), and model III (adjusted for all covariates to rule out possible confounding effects; including model II plus cancer characteristics, referred to as “adjusted for all factors”). Factors associated with depression and anxiety (as dependent variables) (score > 10) were tested via logistic regression analysis, where OR and 95% CI were calculated for each independent variable. A two-tailed p-value of <0.05 was considered statistically significant.

## Results

In total, 121 eligible patients were approached, and 92 patients with various cancer diagnoses agreed to participate in this study. The mean age of the patients was 51.24 ± 15.15 (range: 18-86) years, and approximately one-third (32.6%) of the patients were aged older than 60 years. Among them, 51 (55.4%) patients were women and 41 (44.6%) were men. Most participants (n = 81, 88.0%) were married, and only 11 (12%) participants were single, widow, or divorced. Only 10.9% of patients had a family history of mental disorders. Table 1 shows the sociodemographic characteristics of the participants.

**Table 1:** General characteristics of the patients ^a^: All patients, except five (5.4%), have siblings.

Variables		N = 92	%
Age (years)	Mean ± SD, Min–Max	51.24 ± 15.15, 18–86	
	18–40	24	26.1
	41–60	38	41.3
	>60	30	32.6
Sex	Male	41	44.6
	Female	51	55.4
Social status	Single	11	12.0
	Married ^a^	81	88.0
Family history of mental disorders	Yes	10	10.9
	No	82	89.1

The median cancer duration was six months (IQR: 4-22.5), with a minimum of one month and a maximum of 10 years. Moreover, more than two-thirds (68.5%) of patients had a cancer duration of <1 year.

The most prevalent types of cancer were breast cancer and lymphoma (n = 18, 19.6% each), followed by colorectal cancer (n = 16, 17.4%), bladder and stomach cancer (n = 7, 7.6% each), pancreatic and ovarian cancer (n = 5, 5.4% each), liver cancer (n = 3, 3.3%), prostate cancer (n = 2, 2.2%), and other types of cancer (n = 11, 12.0%). A total of 16 (17.4%) patients had cancer-associated pain. Only one patient (1.1%) presented with early stage I cancer, three (3.3%) with stage II, 24 (26.1%) with stage III, and 64 (69.6%) with stage IV. Approximately 84.8% of patients were receiving chemotherapy alone; 5.4%, both chemotherapy and radiotherapy; and 9.8%, other treatment regimens. Furthermore, 46.7% of the participants received surgical interventions. Table 2 shows the cancer characteristics of the participants.

**Table 2:** Cancer characteristics of the participants ^a^: Including brain/CNS, nasopharyngeal, uterine, thyroid, lung, renal, and other types of cancer.

Variables		N = 92	%
Cancer duration (months)	Median (IQR), Min–Max	6 (4–22.5), 1–120	
	<12	63	68.5
	12–24	19	20.7
	>24	10	10.9
Type of cancer according to the main organ	Breast cancer	18	19.6
	Lymphoma	18	19.6
	Colorectal cancer	16	17.4
	Bladder cancer	7	7.6
	Stomach cancer	7	7.6
	Pancreatic cancer	5	5.4
	Ovarian cancer	5	5.4
	Liver cancer	3	3.3
	Prostate cancer	2	2.2
	Other types ^a^	11	12.0
Cancer-associated pain	Yes	16	17.4
Current treatment	Chemotherapy only	78	84.8
	Chemotherapy and radiotherapy	5	5.4
	Others	9	9.8
Surgical intervention	Yes	43	46.7

According to HADS, the median depression score was 7 (IQR: 1.25-11, range: 0-19). In total, 53 (57.6%) patients had no clinical symptoms of depression. Furthermore, 14 (15.2%) patients presented with mild depression and 25 (27.2%) with symptomatic depression. The median number of patients with anxiety was three (IQR: 1-7, range: 0-18). Additionally, 70 (76.1%) patients had no clinical symptoms of anxiety. Meanwhile, five (5.4%) patients presented with mild anxiety and 17 (18.5%) with symptomatic anxiety. Table 3 shows the depression and anxiety scores of the participants according to HADS.

**Table 3:** Depression and anxiety scores of the participants according to HADS HADS: Hospital Anxiety and Depression Scale

Variables		N = 92	%
Depression score	Median (IQR), Min–Max	7 (1.25–11), 0–19	
	No clinical symptoms of depression (0–7)	53	57.6
	Mild depression (8–10)	14	15.2
	Symptomatic depression (11–21)	25	27.2
Depression score	Median (IQR), Min–Max	3 (1–7), 0–18	
	No clinical symptoms of anxiety (0–7)	70	76.1
	Mild anxiety (8–10)	5	5.4
	Symptomatic anxiety (11–21)	17	18.5

Both depression and anxiety scores were tested against different sociodemographic and cancer characteristics. There were significant associations between depression and marital status (P = 0.032), the presence of cancer-associated pain (P = 0.033), and current treatment with chemotherapy only (P = 0.046). There were no significant associations between depression and age, sex, family history of mental disorders, cancer duration, current cancer stage, and surgical interventions (P > 0.05). There was a significant association between anxiety and the presence of cancer-associated pain (P = 0.001). There were no significant associations between anxiety and age, sex, marital status, family history of mental disorders, cancer duration, current cancer stage, and surgical interventions (P > 0.05).

Table 4 shows the distribution of anxiety and depression in different subgroups.

**Table 4:** Association between depression and anxiety scores and different sociodemographic and cancer characteristics FH: Family history. *: Significant.

Variables		Depression score			Anxiety score		
		Patients without depression, n = 67 (%)	Patients with depression, n = 25 (%)	P-value	Patients without anxiety, n = 75 (%)	Patients with anxiety, n = 17 (%)	P-value
Age (years)	18–40	16 (23.9)	8 (32.0)	0.116	18 (24.0)	6 (35.3)	0.63
	41–60	32 (47.8)	6 (24.0)		32 (42.7)	6 (35.3)	
	>60	19 (28.4)	11 (44.0)		25 (33.3)	5 (29.4)	
Sex	Male	33 (49.3)	8 (32.0)	0.163	33 (44.0)	8 (47.1)	1
	Female	34 (50.7)	17 (68.0)		42 (56.0)	9 (52.9)	
Social status	Single	11 (16.4)	0 (0.0)	0.032*	11 (14.7)	0 (0.0)	0.207
	Married	56 (83.6)	25 (100.0)		64 (85.3)	17 (100.0)	
FH of the mental disorder		10 (14.9)	2 (8.0)	0.502	8 (10.7)	4 (23.5)	0.224
Cancer duration (months)	<12	45 (67.2)	18 (72.0)	0.096	50 (66.7)	13 (76.5)	0.28
	12–24	12 (17.9)	7 (28.0)		15 (20.0)	4 (23.5)	
	>24	10 (14.9)	0 (0.0)		10 (13.3)	0 (0.0)	
Cancer-associated pain		8 (11.9)	8 (32.0)	0.033*	8 (10.7)	8 (47.1)	0.001*
Current stage	Stage I	1 (1.5)	0 (0.0)	0.354	1 (1.3)	0 (0.0)	0.777
	Stage II	1 (1.5)	2 (8.0)		3 (4.0)	0 (0.0)	
	Stage III	19 (28.4)	5 (20.0)		20 (26.7)	4 (23.5)	
	Stage IV	46 (68.7)	18 (72.0)		51 (68.0)	13 (76.5)	
Current treatment	Chemotherapy only	53 (79.1)	25 (100.0)	0.046*	61 (81.3)	17 (100.0)	0.154
	Chemotherapy and radiotherapy	5 (7.5)	0 (0.0)		5 (6.7)	0 (0.0)	
	Others	9 (13.4)	0 (0.0)		9 (12.0)	0 (0.0)	
Surgical intervention		33 (49.3)	10 (40.0)	0.487	37 (49.3)	6 (35.3)	0.42

The association between depression and anxiety scores and different significant variables in the univariate analysis was further explored using both unadjusted and adjusted ORs with 95% CI. Both scores were entered into the model as a dichotomous categorical variable (including patients without or with mild depression or anxiety in the reference group).

A significant association was observed between depression score and social status and the presence of pain, with married patients and those with pain having higher ORs for depression than single ones and those without pain (1.45 (1.25-1.67), P = 0.041 vs. 2.24 (1.17-4.26), P = 0.029). In addition, there was a significant association between anxiety score and the presence of pain, with patients with pain having higher ORs for anxiety than those without (4.22 (1.93-9.26), P = 0.001). After adjusting for all factors in model III, the OR for depression in patients with pain was 4.71 (1.19-18.52), with patients with pain having significantly higher OR than those without (P = 0.027). Similarly, the OR for anxiety in patients with pain was 9.08 (2.75-19.67), with patients with pain having significantly higher ORs than those without (P = 0.002) (Table 5).

**Table 5:** Association between depression and anxiety scores and different study variables OR: Odds ratio; AOR: Adjusted odds ratio. Model-I: Unadjusted. Model II: Adjusted for sociodemographic characteristics and family history of mental disorders. Model III: Adjusted for all factors (model 2 plus cancer characteristics). * Significant.

Variables	Number of cases	Model I OR (95% CI)	Model II AOR (95% CI)	Model III AOR (95% CI)
For depression				
Social status (Single vs Married)	11 vs 81	1.45 (1.25–1.67)	0.71 (0.35–1.41)	0.40 (0.09–1.78)
P-value		0.041*	0.327	0.231
Cancer-associated pain (absent vs. present)	16 vs 76	2.24 (1.17–4.26)	3.64 (1.09–12.11)	4.71 (1.19–18.52)
P-value		0.029*	0.035*	0.027*
Current treatment (others vs chemotherapy only vs chemotherapy and radiotherapy)	9 vs 78 vs 5	1.50 (0.45–5.07) vs 0.84 (0.43–1.76)	1.26 (0.48–7.04) vs 0.79 (0.21–1.99)	1.24 (0.37–4.17) vs 0.71 (0.21–2.35)
P-value		0.804	0.993	0.718
For anxiety				
Cancer-associated pain (absent vs. present)	16 vs 76	4.22 (1.93–9.26)	6.94 (2.82–14.64)	9.08 (2.75–19.67)
P-value		0.001*	0.001*	0.002*

We further analyzed variables in the univariate analysis that significantly affected depression and anxiety scores using the multinomial logistic regression model to explore independent variables associated with depression and anxiety. The presence of pain was significantly associated with depression (OR = 3.47, 95% CI: 1.163-10.62; P = 0.029) and anxiety (OR = 7.44, 95% CI: 1.46-2.11; P = 0.001) in patients with cancer in Najran City (Table 6).

**Table 6:** Multinomial logistic regression analysis of the factors associated with depression and anxiety among patients with cancer in Najran City. OR: Odds ratio, CI: Confidence interval. *: Significant.

Independent variables	Coefficient	OR	95% CI	P-value
For depression				
Social status (married)	0.72	2.06	(0.78–5.42)	0.142
Associated pain (present)	1.24	3.47	(1.163–10.62)	0.029*
Current treatment (chemotherapy only)	0.41	1.45	(0.57–3.69)	0.513
For anxiety				
Associated pain (present)	2.01	7.44	(2.24–14.76)	0.001*

## Discussion

The present study primarily aimed to assess the prevalence of depression symptoms among patients with cancer in Saudi Arabia and their associated risk factors.

Our study found that 42.4% of patients presented with depressive symptoms, 15.2% with mild depression, and 27.2% with symptomatic depression. These results are fairly similar to those reported by Nikbakhsh et al. In their study, 48% of patients presented with depressive symptoms, 26.7% with mild depression, and 21.3% with symptomatic depression [11]. These results did not significantly from those of Ciaramella et al. and Kai-hoi et al., who reported that 28% and 29% of patients with cancer presented with depression. In contrast, our results significantly differed from those of Carroll et al., who reported that 9.9% of patients with cancer had depressive disorders [15,17,27].

The present study showed no significant difference between patients with depression and those without in terms of age, which is in accordance with the results obtained by other researchers [15,17]. However, Nikbakhsh et al. reported contradictory findings; they showed that older patients had a higher frequency of depression than younger patients. Moreover, other studies have revealed that older patients with cancer are less likely to present with depression than younger ones [11,28,29]. Our study found no difference between patients with depression and those without in terms of sex, which is consistent with the results of Ciaramella et al. and Kai-hoi et al. [15,17].

In our study population, there was a significant association between depression and marital status. This unexpected result can be attributed to disintegration and dependence of the family and financial losses, which can increase the risk of psychological issues in patients with cancer. In addition, the high cost of cancer treatment can pose a heavy burden on household economies. The exact level of social support for single and married patients was not assessed in the present study. Theoretically, some single patients might have a sufficient network of friends and relatives [30-32].

Our study found no significant associations between depression and a family history of mental disorders. In addition, a significant association was observed between depression and cancer-associated pain. This is consistent with the results of other studies showing that the pain group had a significantly higher incidence of depressive disorders than the without-pain group [15,33,34]. This finding differed from the findings of Kai-hoi et al., who reported that the nature and severity of pain did not vary between patients with depression and those without [17].

There was a significant association between depression and current treatment with chemotherapy only, which is similar to the findings of Nikbakhsh et al. This could be attributed to the poor end-stage prognosis of the patients, which can only be managed with chemotherapy [11]. Our study found no significant associations between depression and cancer duration, current cancer stage, and surgical interventions.

Our study secondarily aimed to assess the prevalence of depression and anxiety symptoms among patients with cancer in Saudi Arabia and their associated risk factors. Our study found that 23.9% of the patients presented with anxiety symptoms, 5.4% with mild anxiety, and 18.5% with symptomatic anxiety. Our results were similar to those of Van den Brekel et al. and Safaie et al. (22% and 28.6%, respectively) [35,36].

Our findings did not significantly differ from those of Nikbakhsh et al., who revealed that 29.3% of patients with cancer presented with mild anxiety and 16.7% with symptomatic anxiety [11]. However, our findings differ from those of Malekian et al. and Carroll et al. (18.5% and 17.7%, respectively) [16,27]. In the present study, age was not associated with anxiety, similar to a study by Truong et al. but is different from studies that detected a significant association between anxiety and younger age and studies showing a significant association between anxiety and older age [11,12,35].

In our sample, sex was not significantly associated with anxiety, which is in accordance with the findings of Truong et al. [12]. However, this finding differs from that of some other studies that reported a higher prevalence of anxiety in women than in men [16,35,36]. Marital status was not significantly associated with anxiety score, and this finding is similar to that of Truong et al. [12]. Our study found no significant association between anxiety and a family history of mental disorders.

The present study detected a significant association between anxiety and cancer-associated pain. This result is similar to that of other studies [34,37]. Moreover, it is in accordance with the study of Thielking, which showed that patients with cancer who had treatment-related pain were more likely to present with anxiety than those with cancer who did not present with pain [38].

In our study, there was no significant association between anxiety and cancer duration. This result is similar to that of Malekian et al. However, it differs from some studies showing that patients with a longer cancer duration present with less anxiety [12,16].

In our study, there were no significant associations between anxiety and cancer treatment and surgical interventions. These results are similar to those of Malekian et al. but not to those of Nikbakhsh et al., who reported that patients who received a single treatment with chemotherapy had a higher prevalence of anxiety [11,16].

In our study, there was no significant association between anxiety and the current cancer stage. These results are similar to those of Malekian et al. [16]. However, they differ from those of other studies showing that patients with more advanced-stage cancer are more likely to have higher anxiety levels [12,19].

Our study had several limitations. First, clinical structural interviews with patients were not conducted, which led to the diagnosis of depression and anxiety using psychometric tools. A precise diagnosis might have been established if a structured interview was conducted.

## Conclusions

Our study highlighted the importance of identifying depression and anxiety in patients with cancer. Marital status, presence of pain, and history of chemotherapy were significantly associated with depression and pain with anxiety. Nevertheless, further studies with a higher number of patients with cancer should be conducted in Saudi Arabia and other Arab countries. Moreover, ongoing screening for depression and anxiety is an important part of providing quality care for patients with cancer. Appropriate treatment interventions must be provided to patients with cancer presenting with mental disorders.
